# Nailfold capillaroscopy in systemic lupus erythematosus: Correlation with clinical and laboratory findings

**DOI:** 10.1016/j.jdin.2026.06.018

**Published:** 2026-07-07

**Authors:** Supriya Paudel, Mahesh Mathur, Sunil Jaiswal, Shreya Paudel

**Affiliations:** a Department of Dermatology, College of Medical Sciences, Bharatpur, Nepal; b Department of Dermatology, Chitwan Medical College, Bharatpur, Nepal; c College of Biomedical Engineering and Applied Sciences, Kathmandu, Nepal

**Keywords:** nailfold capillaroscopy (NFC), systemic lupus erythematosus (SLE)

Systemic lupus erythematosus (SLE) is a chronic, autoimmune connective tissue disorder characterized by multisystem involvement and a heterogeneous spectrum of clinical manifestations that mainly affects young women. The skin is the second most frequently affected organ after the joints, observed among 85% of patients during the course.^1^ Nailfold capillaroscopy (NFC) has emerged as a noninvasive, easy-to-perform bedside procedure to detect early peripheral microvascular changes in connective tissue diseases, including SLE, with both diagnostic and prognostic value.^2^^,^^3^ This study aims to bridge the gap of most existing literature from Western or East Asian cohorts, with relatively fewer studies from South Asia, by evaluating the pattern and frequency of NFC in SLE and correlating their associations with clinical and laboratory parameters.

This hospital-based, observational cross-sectional study included 60 patients with SLE and mucocutaneous lesions who presented from February 2023 to July 2025. The exclusion criteria included overlap syndrome, diabetes mellitus, hypertension, pregnancy, smoking, and chronic infections because detected microangiopathic changes can affect the results. A detailed history and physical examination were performed, and all were evaluated for Raynaud phenomenon (RP) (Supplementary Table I, available via Mendeley at https://data.mendeley.com/datasets/9tbbgbw5xc/1). Hematological and immunological laboratory parameters including complement C3/C4 and 24-hour urine protein were obtained.

NFC was performed using a Firefly DE300 handheld digital dermoscope (Firefly Global) on the fourth and fifth fingers of both hands after 15 to 20 minutes of acclimatization at 22 to 25 °C.^4^ The capillaroscopic patterns were categorized as normal, SLE, scleroderma/dermatomyositis, or nonspecific, and they were reviewed independently by 2 dermatologists (SP and SJ). The normal pattern was defined by regular capillary distribution with normal density and morphology. SLE pattern included dilated capillary loops, tortuosity, and microhemorrhages without significant capillary loss. Scleroderma/dermatomyositis pattern was characterized by giant capillaries, capillary dropout, and disorganization. Nonspecific pattern comprised mild dilatation or tortuosity not fulfilling criteria for defined connective tissue disease patterns. Statistical analysis was performed using SPSS version 22.0, and categorical variables were compared using the χ^2^ test, with *P* < .05 considered significant.

Among 60 patients, 95% were women with a mean age of 29.6 ± 11.4 years. Photosensitivity was reported in 45% and RP in 56.6%. Additional clinical findings are shown in Supplementary Figures 1 to 4 (available via Mendeley at https://data.mendeley.com/datasets/9tbbgbw5xc/1). Serologically, 76.6% had high antinuclear antibody titers (>1:160), 18.3% had low complement levels, 36.6% were positive for anti-double stranded DNA, 25% were positive for anti-Smith, and 8.3% had antiphospholipid antibodies. Abnormal hematological parameters were: elevated erythrocyte sedimentation rate (63.3%), anemia (31.6%), leukopenia (26.6%), thrombocytopenia (25%), and 24-hour proteinuria (50%) (Supplementary Table II, available via Mendeley at https://data.mendeley.crom/datasets/9tbbgbw5xc/1).

Abnormal NFC morphology was prevalent among 45% of patients; SLE, scleroderma, and nonspecific patterns were observed in 31.6%, 3.3%, and 10%, respectively (Fig 1 and Supplementary Figs 5 and 6, available via Mendeley at https://data.mendeley.com/datasets/9tbbgbw5xc/1). NFC changes were significantly associated with RP (*P* = .014), anti-double stranded DNA positivity (*P* = .027), and proteinuria (*P* = .020) (Table 1). Similarly, malar rash correlated with high antinuclear antibody titers (*P* = .043), and vasculitic lesions correlated with hypocomplementemia (*P* = .001) significantly.

**Fig 1:**
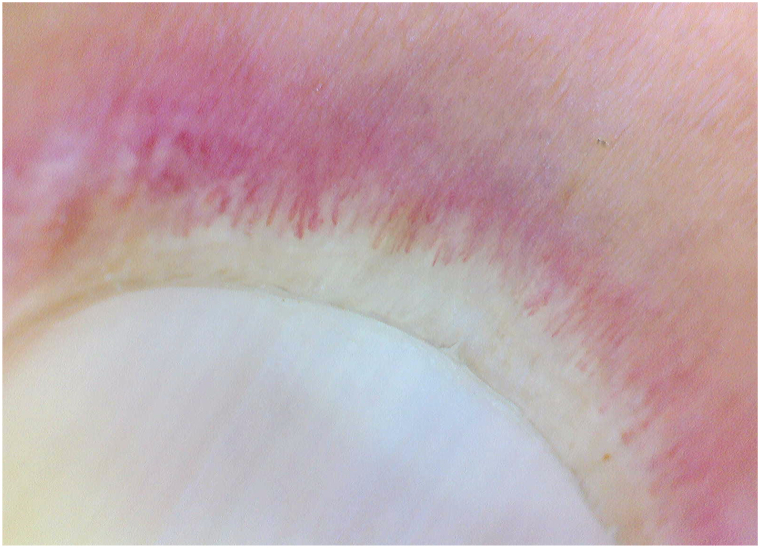
SLE pattern demonstrating dilated and tortuous capillary loops with microhemorrhages and preserved capillary density. *SLE*, Systemic lupus erythematosus.

**Table I:** Pattern of nailfold capillaroscopy in SLE depicted as number and percentage and association with cutaneous and laboratory parameters (χ^2^ test applied and *P* value calculated)

Study patients	Normal NFC (n/%)	SLE pattern (n/%)	Scleroderma pattern (n/%)	Nonspecific pattern (n/%)	*P* value
Raynaud phenomenon (*n =* 34)	14/41.1	9/26.4	3/8.82	8/23.5	.014∗
Anti-double stranded DNA (*n =* 22)	8/36.3	7/31.8	1/4.5	6/27.2	.027∗
24-hour proteinuria (*n =* 30)	12/40	11/36.6	2/6.6	5/16.6	.020∗
Antinuclear antibody (*n =* 46)	24/52.1	11/23.9	3/6.5	8/17.3	.425
APLA (*n =* 5)	4/80	0	0	1/20	.241
Anti-Smith (*n =* 15)	7/46.6	4/26.6	2/13.3	2/13.3	.454
Erythrocyte sedimentation rate (*n =* 35)	17/48.5	8/22.8	2/5.7	8/22.8	.236

*APLA*, Antiphospholipid antibodies; *NFC*, Nailfold capillaroscopy; *SLE*, systemic lupus erythematosus.

∗ Values are statistically significant.

In this cohort, nearly half of the patients exhibited NFC abnormalities, which correlated with RP, anti-double stranded DNA positivity, and proteinuria. These findings suggest that NFC abnormalities may reflect underlying microvascular involvement and systemic disease activity in hospitalized patients with SLE. Therefore, NFC may be considered a noninvasive, adjunctive assessment tool in the evaluation of SLE; however, its role in early detection of vasculopathy or risk stratification cannot be established from this study and warrants validation through larger, longitudinal studies.^3^^,^^5^

## Conflicts of interest

None disclosed.
